# Therapeutic, preventive, and pathogenetic advances in viral hepatitis

**DOI:** 10.3389/fcimb.2026.1939337

**Published:** 2026-09-18

**Authors:** Hong Jiang, Yi-fan Zhang, Ru-chao Zhang, Ya-nan Xing

**Affiliations:** Department of Gastrointestinal Surgery, Affiliated Central People’s Hospital, Yichang Central People’s Hospital, Three Gorges University, Yichang, China

**Keywords:** diagnosis, epidemiology, infection, molecular characteristics, viral hepatitis

## Abstract

Viral hepatitis and associated liver injury represent a major global public health concern, capable of progressing to chronic liver disease, cirrhosis, and hepatocellular carcinoma. This review systematically summarizes classic hepatotropic viruses—including hepatitis B virus (HBV), hepatitis C virus (HCV), and hepatitis A virus (HAV)—as well as atypical liver-injury pathogens such as hepatitis E virus (HEV), hepatitis D virus (HDV), human cytomegalovirus (HCMV), Epstein–Barr virus (EBV), herpes simplex virus (HSV), and yellow fever virus, with respect to their molecular biological characteristics, global epidemiological profiles, infection and pathogenic mechanisms, and strategies for diagnosis, treatment, and prevention. Particular emphasis is placed on the genomic architecture and replication cycles of these viruses, host immune responses and viral evasion strategies, key molecular events driving disease progression, and recent advances and remaining challenges in vaccination, direct-acting antiviral therapy, and public health interventions. By integrating evidence across multiple pathogens, this review aims to provide a systematic theoretical reference for the precise prevention and control of viral hepatitis and associated liver injury, as well as for the optimization of clinical diagnosis and management.

## 1 Introduction

### 1.1 Viral hepatitis

Viral hepatitis represents a systemic infectious disease primarily characterized by hepatic injury, caused by five hepatotropic viruses (hepatitis A, B, C, D, and E viruses), with global endemicity resulting in approximately 1.4 million deaths annually—comparable to the mortality burden of tuberculosis and HIV/AIDS (Cai et al., 2026). Among these, hepatitis B virus (HBV) and hepatitis C virus (HCV) possess the capacity to establish chronic infections, affecting an estimated 240 million and 71 million individuals worldwide, respectively. In the absence of therapeutic intervention, 15%–30% of chronically infected patients progress to cirrhosis or hepatocellular carcinoma, accounting for 78% of global hepatocellular carcinoma cases and 57% of cirrhosis-related morbidity. Through implementation of immunization programs, China has successfully reduced hepatitis B surface antigen (HBsAg) seroprevalence to below 1% among children under five years of age; however, the overall population HBsAg positivity rate remains at 7.18%, with approximately 93 million chronic HBV carriers.

Beyond classical hepatotropic viruses, atypical hepatotropic viruses—including cytomegalovirus (CMV), Epstein-Barr virus (EBV), herpesviruses, and yellow fever virus—can induce acute or chronic hepatocellular injury under specific clinical circumstances (Wang et al., 2026). CMV typically manifests as subclinical infection in immunocompetent hosts, with only approximately 10% developing mild-to-moderate transaminase elevation; jaundice remains exceedingly rare. Conversely, in immunocompromised patients—such as organ transplant recipients, individuals receiving high-dose corticosteroid therapy, or those with HIV infection—invasive hepatitis may develop, characterized by cholestasis, granulomatous hepatitis, and potentially acute hepatic failure. During primary infection or viral reactivation, EBV demonstrates elevated transaminases in 40–90% of cases, with 5–10% developing jaundice; progression to acute hepatitis or fulminant hepatic failure occurs rarely. Yellow fever virus predominantly causes hepatotropic hemorrhagic fever, with early infection demonstrating significant hepatocellular necrosis, marked elevations of aspartate aminotransferase (AST) and alanine aminotransferase (ALT) (10–50-fold above normal), pronounced hyperbilirubinemia, and coagulopathy; severe cases may progress to acute hepatic failure (Damas et al., 2026). Nevertheless, compared to classical hepatotropic viruses, atypical hepatotropic viruses demonstrate substantial variability in incidence and clinical severity among general populations.

The World Health Organization has established targets to reduce new viral hepatitis infections by 90% and mortality by 65% by 2030. Achievement of these objectives necessitates comprehensive strategies encompassing vaccination programs, blood safety protocols, safe injection practices, prevention of mother-to-child transmission, and systematic screening with standardized antiviral therapy for chronically infected individuals.

## 2 Hepatitis B virus

Hepatitis B virus (HBV) represents the sole known DNA virus capable of establishing chronic hepatitis in humans. Following infection, the virus can persist within hepatic tissue for years or even lifelong, inducing chronic inflammatory necrosis and fibrogenesis (Xu et al., 2014). In the absence of clinical intervention, this pathological process may progressively advance to cirrhosis, hepatic failure, or primary hepatocellular carcinoma, rendering HBV one of the principal etiologies necessitating long-term surveillance and management in patients with chronic liver disease.

### 2.1 Molecular characteristics of HBV

The genome of hepatitis B virus (HBV) consists of a partially double-stranded relaxed circular DNA (rcDNA) approximately 3.2 kb in length, characterized by a complete negative strand and a gapped positive strand (Tsukuda and Watashi, 2020). The circular conformation is maintained through covalent linkage of the viral polymerase and residual 5’-terminal protein (Debsikréo et al., 2025). Following viral entry into hepatocytes, rcDNA is transported to the nucleus and converted to covalently closed circular DNA (cccDNA) via host DNA repair enzymes (Elizalde et al., 2022). cccDNA exists stably in the form of a minichromosome and serves as the transcriptional template for all viral RNAs, including the 3.5 kb pregenomic RNA (pgRNA). HBV represents the only DNA virus that relies on an RNA intermediate for DNA replication: in the cytoplasm, pgRNA, together with viral polymerase (P protein), is encapsidated by newly synthesized core protein (HBcAg) to form nucleocapsids (Wang et al., 2022). Within these capsids, P protein first reverse transcribes the negative-strand DNA using pgRNA as template, subsequently synthesizing positive-strand DNA using the negative strand as template, ultimately generating new rcDNA molecules. Owing to the absence of proofreading activity during this reverse transcription process, HBV exhibits a nucleotide misincorporation rate of 1.4–3.2×10^-5^ per replication cycle, facilitating rapid accumulation of quasispecies diversity and providing the molecular basis for immune evasion, antiviral drug resistance, and vaccine breakthrough (Kao, 2002).

### 2.2 Epidemiology of HBV

The epidemiological landscape of hepatitis B virus (HBV) infection demonstrates a distinct tripolar global distribution: the Western Pacific, African, and Southeast Asian regions collectively harbor approximately 85% of global chronic infections (Ezzikouri et al., 2013; Assefa et al., 2024). Updated 2023 WHO data indicate that 296 million individuals worldwide are living with chronic HBV infection, with 1.2 million new infections and 820,000 deaths annually—accounting for 83% of all viral hepatitis mortality and representing the leading cause of death from a single infectious agent (WHO, 2023) (Liu and Kao, 2007). HBsAg seroprevalence varies markedly by region: 5.9% in the Western Pacific, 8.4% in Africa, and merely 1.5% in Europe (Ciommo et al., 1993). At the national level, 11 countries including Mongolia, Solomon Islands, and Moldova exhibit HBsAg positivity rates ≥10%, constituting hyperendemic zones (Baudi et al., 2017).

Age represents a critical determinant of epidemic patterns: in Africa, HBsAg seroprevalence in children under five years remains above 3%, indicating persistent active mother-to-child transmission; conversely, in Western Europe, incident infections among adults predominantly affect sexually active individuals aged 20–34 years and injection drug users, with chronicity rates <1% (Ott et al., J Hepatol, 2021). Gender disparities are also pronounced, with global HBsAg positivity rates of 4.8% in males versus 3.1% in females; age-standardized mortality from HBV-related hepatocellular carcinoma in males is 2.4-fold higher than in females (Baudi et al., 2017).

In China, population-wide HBsAg seroprevalence was 9.75% in 1992. Through implementation of the “first dose within 24 hours of birth plus two subsequent doses” vaccination strategy and, since 2012, antiviral maternal-infant blocking for pregnant women with high viral loads, HBsAg positivity in children under five years decreased to 0.3% by 2020—achieving the WHO Western Pacific regional target of <1% ahead of schedule. However, the absolute disease burden remains substantial, with 75–86 million existing chronic infections (28% of global total), of whom only 22% have been diagnosed and 18% are receiving treatment, indicating significant gaps toward the WHO 2030 elimination targets of 90% diagnosis and 80% treatment coverage (Cui et al., China CDC Weekly, 2022). Modeling studies project that maintaining current intervention intensity will result in 420,000 HBV-related annual deaths in China by 2030, representing a 14% increase from 2020; conversely, expanding adult screening to 90% and treatment coverage to 80% could prevent 3.3 million chronic infections, 450,000 cases of cirrhosis, and 350,000 hepatocellular carcinoma deaths before 2030, generating net healthcare cost savings of $18.5 billion USD (Wang et al., 2014).

In the African region, 2023 coverage with three-dose neonatal vaccination reached only 72%, substantially below the global 90% target. Combined with inadequate control of unsafe injections, sexual transmission, and iatrogenic exposures, HBV-related mortality in Africa is projected to increase by a mean annual rate of 2.8% during 2025–2035, representing the most significant gap in global hepatitis elimination efforts (Wang et al., 2014; Baudi et al., 2017).

Collectively, HBV epidemiology has transitioned from a phase of “high endemicity, low intervention” to “moderate-low endemicity, high residual burden.” Whether the transmission chain can be interrupted through large-scale screening, universal vaccination, and expanded antiviral therapy over the coming decade will determine the success of achieving WHO’s 2030 goal of eliminating viral hepatitis as a public health threat.

### 2.3 The infection and pathogenic mechanisms of HBV

Hepatitis B virus (HBV) infection initiates through high-affinity binding of mature Dane particles containing envelope proteins (HBsAg) to the sodium-taurocholate cotransporting polypeptide (NTCP) on the basolateral membrane of hepatocytes, followed by clathrin-mediated endocytosis into the cytoplasm (Seeger and Mason, 2015). Upon fusion of the viral envelope with endosomal membranes, partially double-stranded relaxed circular DNA (rcDNA) is released into the cytoplasm and translocated to the nucleus together with viral core protein (HBcAg) capsids (Poovorawan et al., 2025). Within the nucleus, host DNA repair enzymes ligate the gap in rcDNA and remove covalently attached viral polymerase, converting it to covalently cccDNA. cccDNA exists stably in the form of a minichromosome, remains unaffected by antiviral agents directly, and constitutes the molecular foundation for persistent HBV infection and post-treatment relapse (Zhang et al., 2021).

At the pathogenic level, HBV itself is non-cytopathic; hepatocellular injury is predominantly driven by virus-specific cytotoxic T lymphocytes (CTLs) and subsequent inflammatory cascade reactions (Thomas, 1990; Bar-Yishay et al., 2011). During acute self-limited infection, polyclonal, multispecific CD8^+^ T cells secrete IFN-γ and TNF-α, inducing apoptosis of infected hepatocytes and activating non-cytolytic antiviral pathways to achieve viral clearance. In chronic infection, however, T cell exhaustion induced by circulating HBeAg and abundant soluble HBsAg, expansion of regulatory T cells (Tregs), and upregulation of PD-1/CTLA-4 immune checkpoints result in CTL dysfunction, precluding effective cccDNA elimination and permitting persistent viral replication. Concurrently, HBV actively suppresses innate immunity through multiple mechanisms: viral polymerase disrupts RIG-I-MAVS interactions, inhibiting IRF3 phosphorylation and thereby downregulating type I interferons; HBx protein degrades mitochondrial antiviral signaling protein (MAVS) and the SMC5/6 complex, attenuating cellular viral DNA sensing and enhancing cccDNA transcriptional activity; N^6^-methyladenosine (m^6^A) modification of HBV RNA facilitates evasion from RIG-I immune sensor recognition while promoting nuclear export and translation of viral transcripts, further amplifying viral protein expression (Jeng et al., 2025).

Prolonged chronic inflammation drives repeated hepatocyte necrosis-regeneration cycles accompanied by oxidative stress and chromosomal instability; 30%–40% of chronically infected individuals progress to hepatic fibrosis and cirrhosis within 20–30 years, ultimately developing hepatocellular carcinoma through cccDNA integration into the host genome and HBx-mediated transcriptional dysregulation leading to inactivation of tumor suppressor genes including p53 and RB. Although integrated fragments cannot support complete viral replication, they continuously express truncated surface proteins and HBx, establishing an immune evasion state characterized by “surface antigen secretion with persistent low-level viral replication,” rendering clinical cure substantially more challenging (Xiang and Xie, 2015; Deng and Lu, 2016).

In summary, HBV achieves persistent infection and progressive liver disease through multiple strategies including establishment of cccDNA reservoirs, regulation of RNA modifications, interference with innate immune signaling, and induction of T cell exhaustion—presenting formidable challenges for antiviral therapy and functional cure.

### 2.4 The diagnosis, treatment, and prevention of HBV

The diagnosis, treatment, and prevention of hepatitis B virus (HBV) infection have established a comprehensive closed-loop system encompassing high-sensitivity laboratory detection, potent low-resistance antiviral therapy, universal vaccine coverage and zero mother-to-child transmission blocking (Mahoney, 1999).

Serological markers remain the cornerstone of initial screening: HBsAg positivity indicates active infection, with persistence ≥6 months establishing chronicity; anti-HBs positivity denotes protective immunity; HBeAg/anti-HBe seroconversion reflects changes in viral replication levels; total anti-HBc positivity indicates past or present infection, while IgM anti-HBc serves to differentiate acute infection. However, serology cannot quantify viral load; high-sensitivity real-time quantitative PCR has replaced traditional hybridization methods as the gold standard for HBV DNA detection, informing treatment initiation, efficacy monitoring, and resistance surveillance (Mak et al., 2023). For patients with HBsAg seroclearance but persistent hepatic dysfunction, cccDNA-targeted digital PCR or *in situ* hybridization of liver tissue may be employed to confirm residual viral templates; serum hepatitis B core-related antigen (HBcrAg) and HBV RNA assays provide additional replication activity information when DNA falls below detection limits. Imaging and non-invasive fibrosis assessment facilitate disease staging, with liver biopsy reserved for definitive grading of necroinflammatory activity and fibrosis when clinically indicated (Huang et al., 2025).

For resistant patients, early-generation L-nucleoside resistance may be managed by switching to or adding TDF/TAF; multidrug resistance warrants ETV+TDF/TAF combination therapy (Dény and Zoulim, 2010). Discontinuation criteria are stringent: HBeAg-positive patients require ≥12 months consolidation with undetectable HBV DNA; HBeAg-negative patients should maintain long-term therapy unless HBsAg clearance is achieved. Emerging therapeutics include RNA interference (siRNA), capsid assembly modulators (CAMs), HBsAg release inhibitors (REP 2139), and therapeutic vaccines; clinical trials demonstrate HBsAg decline ≥1 log in some participants, though standalone clearance rates remain limited, suggesting future combination with NAs or IFN for clinical cure (Michel et al., 2011).

Expanded neonatal vaccine coverage represents the central preventive strategy. WHO advocates ≥90% first dose within 24 hours of birth, ≥95% three-dose series completion, including premature infants with birth weight ≥2 kg in routine immunization schedules (Krugman, 1975). For infants born to HBsAg-positive mothers, combined immunoprophylaxis with vaccine and hepatitis B immunoglobulin (HBIG) is supplemented by maternal TDF antiviral therapy initiated at gestational weeks 24–28 for pregnant women with HBV DNA ≥2×10^5^ IU/mL, continuing postpartum through 4–12 weeks, reducing mother-to-child transmission rates to <0.3% (Li et al., 2022). Adult high-risk populations should undergo screening with periodic follow-up; those with antibody titers <10 IU/L may receive 1–3 additional doses. Regarding blood safety, global implementation of 100% voluntary non-remunerated blood donation and nucleic acid amplification technology (NAT) screening has shortened the window period to 6–10 days; unsafe injection rates have declined from 40% in 2000 to 5% in 2020, though certain regions in the Middle East and South Asia remain >15%, necessitating continued promotion of auto-disable syringes (López-Serrano et al., 2013).

## 3 Hepatitis C virus

Hepatitis C virus (HCV) is a blood-borne pathogen that primarily targets hepatocytes, with chronic infection characterized by persistent yet frequently asymptomatic hepatic inflammation that may progressively evolve over decades into advancing fibrosis, cirrhosis, and end-stage liver disease, while significantly increasing the risk of hepatocellular carcinoma. Through high mutation rates that enable evasion of host immune surveillance, the virus establishes a prolonged and refractory persistent state that is difficult to resolve spontaneously, constituting a substantial component of the global burden of chronic liver disease (Yang and Zhu, 2015).

### 3.1 Molecular characteristics of HCV

The genome of HCV consists of a single positive-strand RNA approximately 9.6 kb in length, flanked by 5′ and 3′ UTRs at either terminus, with a single ORF in between (Mahmoudvand et al., 2019). The ORF is translated into a polyprotein precursor of approximately 3,000 amino acids, which is subsequently cleaved by coordinated action of host signal peptidases and viral proteases into at least 10 mature proteins, categorized functionally as structural proteins (Core, E1, E2) and non-structural proteins (p7, NS2, NS3, NS4A, NS4B, NS5A, NS5B) (Dubuisson, 2007). The Core protein binds viral RNA to form the nucleocapsid; E1/E2 glycoproteins are embedded in the lipid envelope and determine host-specific viral entry; p7 forms an ion channel that modulates the pH environment for virion maturation; NS2 possesses intrinsic protease activity responsible for cleavage at the NS2-NS3 junction; NS3 functions as both a serine protease (N-terminal one-third) and RNA helicase (C-terminal two-thirds), participating in downstream polyprotein processing and RNA duplex unwinding; NS4A serves as a cofactor for NS3 protease, enhancing its catalytic efficiency; NS4B induces reticular reorganization of the endoplasmic reticulum membrane, constructing the “membranous web” replication factory; NS5A acts as a scaffold protein for the RNA replication complex, regulating viral assembly and lipid metabolism; NS5B functions as an RNA-dependent RNA polymerase (RdRp), completing genome amplification through a negative-strand intermediate mechanism (Giannini and Bréchot, 2003; Zhang et al., 2024).

Due to the absence of proofreading activity, RdRp introduces nucleotide misincorporations at a rate of approximately 10^-^³–10^-4^ per replication cycle, enabling HCV to rapidly generate a highly genetically heterogeneous quasispecies cloud within the same host, presenting challenges for immune evasion, vaccine development, and resistance to direct-acting antivirals (DAAs) (Cheikhrouhou et al., 2015; Wassenaar et al., 2020). To date, seven genotypes (1–7) and over 90 subtypes have been identified, with inter-genotypic whole-genome divergence ≥30% and substantial diversity within genotypes due to quasispecies dynamics. Viral particles are spherical, 40–60 nm in diameter, enveloped by a host-derived lipid bilayer membrane with surface spikes composed of E1/E2 heterodimers; the nucleocapsid, approximately 33 nm in diameter, consists of Core protein-encapsidated RNA arranged in icosahedral symmetry. The entire replication cycle occurs exclusively in the cytoplasm without DNA intermediate formation, thereby precluding integration into the host genome; however, this absence of stable integrated forms necessitates continuous active RNA replication and protein expression to maintain the infectious state (Pham et al., 2008; Wan et al., 2026).

### 3.2 Epidemiology of HCV

HCV exhibits global endemicity with pronounced regional heterogeneity (Pozzato et al., 2017). WHO 2023 estimates indicate approximately 58 million chronic infections worldwide, with 290,000 annual deaths, 75% concentrated in low- and middle-income countries; the Eastern Mediterranean and European regions demonstrate the highest anti-HCV seroprevalence (2.3% and 1.5%, respectively), while the Western Pacific and Americas show the lowest rates (0.5% and 0.7%) (Pozzato et al., 2017). At the national level, Egypt previously exhibited the world’s highest prevalence (18%–22% in persons aged 15–59 years) due to reuse of non-sterile syringes during schistosomiasis treatment campaigns from the 1950s–1980s; China, with an estimated 40 million infections in 1992 based on serosurveillance, has reduced anti-HCV seroprevalence in persons aged 1–59 years to 0.43% by 2020 through implementation of safe blood transfusion practices, universal disposable syringe promotion, and improved DAA accessibility post-2014, with 5.6–10 million existing infections currently representing the second-largest national burden globally (Ebeling, 1998; Wang, 2013).

Genotype distribution demonstrates geographic clustering: genotype 1b predominates in Europe, North America, and East Asia (50%–70%), genotype 3 is prevalent in South Asia and Northern European injection drug user populations, genotype 4 concentrates in the Middle East and Central Africa, while genotypes 5 and 6 are restricted to localized regions of South Africa and Southeast Asia; distinct genotypes exhibit differential disease progression kinetics and treatment responsiveness, with genotype 3 demonstrating increased steatosis susceptibility and 1.8-fold elevated hepatocellular carcinoma risk (Samimi-Rad et al., 2012). Age and sex stratified curves reveal substantially higher adult infection rates compared to children, peaking at ages 50–59 years (0.77%), with males demonstrating higher seroprevalence than females due to increased exposure through substance use and traumatic medical procedures; urban-rural disparities persist, with anti-HCV positivity in western and rural China approximately twice that of eastern urban areas, attributable to delayed implementation of blood collection and supply supervision and inadequate sterilization of medical instruments at primary healthcare levels (Rossi et al., 2015).

Transmission modalities have evolved temporally: in developed countries, intravenous drug use, men who have sex with men, and tattooing/body piercing account for >60% of incident cases; in developing countries, iatrogenic exposure predominates, with unsafe injections, hemodialysis, and reuse of medical devices contributing to approximately 60% of infections. Given that 55%–85% of acute infections are asymptomatic, global diagnostic coverage remains at merely 36%, with substantial occult reservoirs perpetuating transmission; modeling projections indicate that without expanded screening and treatment, HCV-related cirrhosis and hepatocellular carcinoma mortality will continue increasing at a mean annual rate of 1.8% during 2025–2035, significantly deviating from WHO’s 2030 elimination targets (Shustov et al., 2005; Pino et al., 2013).

### 3.3 The infection and pathogenic mechanisms of HCV

The infection and pathogenesis of HCV constitute a dynamic landscape includes viral entry, intracellular replication, persistence, immune evasion, chronic hepatic injury (Chigbu et al., 2019). Initially, following hematogenous dissemination to the liver, HCV envelope glycoproteins E1/E2 sequentially engage host heparan sulfate proteoglycans, low-density lipoprotein receptor, and scavenger receptor B1, subsequently achieving high-affinity engagement with tetraspanin CD81 and tight junction protein claudin-1 to complete “molecular zipper”-type docking; throughout this process, the virus-host complex undergoes clathrin-mediated endocytosis into early endosomes, with E1/E2-mediated membrane fusion releasing the single-stranded positive-sense RNA genome into the cytoplasm (Adinolfi et al., 2014). The cytoplasmic RNA directly initiates cap-independent translation on the membranous web, generating a ~3,000 amino acid polyprotein precursor that undergoes hierarchical cleavage by host signal peptidases and viral NS2-NS3 and NS3-NS4A proteases to produce 10 mature proteins; among these, NS5B RNA-dependent RNA polymerase conducts replication through a negative-strand RNA intermediate mechanism, NS5A provides the replication complex scaffold, and NS4B induces endoplasmic reticulum vesiculation to construct the viral factory (Żychowska et al., 2024). Due to the absence of proofreading activity in RdRp, replication proceeds with misincorporation rates of 10^-^³–10^-4^, enabling HCV to rapidly generate a highly genetically heterogeneous quasispecies cloud within the same host, establishing the molecular foundation for subsequent immune evasion and drug resistance mutations (Woo and Choi, 2024).

At the innate immunity level, HCV employs multiple strategies to silence host alarm systems: the NS3/4A serine protease specifically cleaves MAVS and TRIF, disrupting RIG-I/MDA5-mediated type I interferon signaling; NS5A competes with TBK1 for binding, inhibiting IRF3 phosphorylation; E2 glycoprotein high-mannose glycans bind DC-SIGN, suppressing dendritic cell maturation. Consequently, interferon levels remain suppressed during early infection, permitting viral establishment of a replication bridgehead (Lonardo et al., 2014). During the adaptive immune phase, although the host generates polyclonal, multi-epitope CD8^+^ CTL responses, high-frequency quasispecies mutations rapidly abrogate individual CTL epitopes; simultaneously, selective variation occurs in the NS3 region Th epitope during chronic infection, eliminating the capacity to stimulate T cell proliferation and resulting in disruption of T helper cell signaling with diminished antibody neutralization efficacy (Zeisel et al., 2015). Notably, HCV infection is not restricted to the liver; viral proteins and RNA can undergo low-level replication or persistence in extrahepatic mononuclear cells, neurons, and vascular endothelium, inducing extrahepatic manifestations including cryoglobulinemia, B-cell non-Hodgkin lymphoma, and atherosclerosis—reflecting the systemic pathogenic characteristics of virus-immune-metabolism cross-regulation (González-Gallego et al., 2011; Felmlee et al., 2013).

Collectively, HCV establishes chronic low-grade inflammatory environments through strategies of precise invasion, efficient replication, dynamic variation along with multi-layered immune evasion, persistently activating yet partially suppressing host immunity, gradually remodeling hepatic architecture and driving end-stage liver disease development.

### 3.4 The diagnosis, treatment, and prevention of HCV

The diagnosis, treatment, and prevention of hepatitis C virus (HCV) infection have entered a new era characterized by precision, efficiency, and elimination potential; however, three critical gaps persist globally: low case detection rates, inadequate treatment coverage, and ongoing risks of reinfection (Omran et al., 2018; Gordon et al., 2019). The diagnostic strategy follows a three-step approach: Step one involves serological screening, primarily utilizing third-generation enzyme-linked immunosorbent assay (ELISA) for anti-HCV antibody detection, with specificity >99%, suitable for large-scale screening, though with a window period of approximately 50–70 days and inability to distinguish past from current infection. Step two comprises nucleic acid testing, employing high-sensitivity real-time quantitative RT-PCR or transcription-mediated amplification (TMA) technology, capable of detecting HCV RNA 7–14 days post-infection with quantitative lower limits ≤15 IU/mL, serving as the gold standard for definitive diagnosis, genotyping, and therapeutic efficacy assessment. Step three involves core antigen (HCV-cAg) testing, which correlates positively with RNA viral load, detectable at 12–15 days, with specificity and sensitivity both >95%, at approximately half the cost of RNA testing—designated by WHO as an alternative strategy for resource-limited settings for early screening, treatment monitoring, and differentiation between past and current infections. For high-risk populations including hemodialysis patients, intravenous drug users, and HIV-coinfected individuals, a combined antibody-antigen-RNA strategy is recommended to maximally shorten the window period and reduce false negatives (Chevaliez and Pawlotsky, 2007; Wilkins et al., 2010).

At the therapeutic level, direct-acting antivirals (DAAs) have completely supplanted the interferon era, achieving a paradigm shift toward all-oral, pan-genotypic, short-duration, high-cure-rate regimens (Poe et al., 2018). Regarding prevention and control, WHO has proposed the ambitious goal of eliminating viral hepatitis as a public health threat by 2030, with core targets of 90% reduction in new infections, 65% reduction in mortality, ≥90% diagnosis, and ≥80% treatment coverage (Duncan and Younossi, 2003). Technological innovations are also accelerating elimination progress: dried blood spot (DBS) sampling combined with portable PCR instruments enables community, prison, and rural health clinics to obtain RNA results within 30 minutes, shortening the diagnosis-to-treatment cascade to less than 24 hours, providing instrumental support for test-and-treat strategies (Winetsky et al., 2020).

Nevertheless, significant gaps remain toward 2030 elimination. Global diagnostic coverage stands at merely 36%, treatment coverage at 20%, with approximately 1.5 million new cases annually—roughly equivalent to cured cases—establishing a cure-reinfection equilibrium (Omran et al., 2018; Winetsky et al., 2020).

Only through establishing a long-term mechanism of government leadership, interdepartmental collaboration, and societal participation can the elimination targets of 90% reduction in new infections and 65% reduction in mortality be achieved within the global 2030 countdown, rendering hepatitis C the first chronic viral infection for which a cure can be achieved in >95% of patients with short-course oral regimens.

## 4 Hepatitis A virus

Hepatitis A virus (HAV) belongs to the genus Hepatovirus within the family Picornaviridae transmitted primarily through the fecal-oral route and represents the sole hepatitis virus known to infect humans exclusively (Feinstone, 2019). Following infection, the incubation period typically ranges from 15 to 50 days, with clinical manifestations varying from asymptomatic infection to acute hepatitis; common symptoms include fever, fatigue, nausea, and jaundice. Notably, elderly patients often demonstrate more severe clinical manifestations compared to children and younger adults. Fortunately, effective inactivated and live-attenuated vaccines are currently available for prevention, with some countries having incorporated these vaccines into routine childhood immunization programs. Recovery from infection generally confers lifelong protective immunity, with reinfection being exceedingly rare.

### 4.1 Molecular characteristics of HAV

The virion of HAV exhibits icosahedral symmetry with a diameter of approximately 27–32 nm and lacks an envelope (Cristina and Costa-Mattioli, 2007). The viral capsid consists of 60 protomers, each containing one molecule each of the three major structural proteins VP1, VP2, and VP3, along with a small amount of VP4 protein (Ticehurst et al., 1983). Cryo-electron microscopy structural analysis reveals that VP1 proteins arrange around the fivefold axes of the icosahedron, while VP2 and VP3 form the interfaces between hexamers and pentamers. Recent studies have demonstrated that the C-terminus of VP1 protein exhibits high heterogeneity, which is essential for viral maturation.

The HAV genome comprises a single positive-strand RNA approximately 7.5 kb in length, terminating with a polyadenylate tail (Kwon et al., 2000; Zhang et al., 2025). The genome contains a 5’-untranslated region (5’UTR), a single open reading frame (ORF), and a 3’-untranslated region (3’UTR). The 5’UTR spans approximately 734 nucleotides and harbors an internal ribosome entry site (IRES), classified as picornavirus type III IRES, responsible for initiating translation of viral proteins (Endo et al., 2007). Both the 3’UTR and poly(A) tail play important regulatory roles in viral replication. Notably, the HAV IRES possesses hepatocyte-specific factor-binding characteristics, contributing to its hepatotropism.

The unique long open reading frame of the viral genome encodes a polyprotein precursor P1-P2-P3 of approximately 2,227 amino acids, which is cleaved by the combined action of viral and cellular proteases into 11 mature proteins (Chávez et al., 2001; Endo et al., 2007; Feinstone, 2019). HAV exhibits distinctive features in molecular evolution (Kwon et al., 2000; Chávez et al., 2001). Its quasispecies diversity during RNA replication is lower than that of other picornaviruses, demonstrating relatively high sequence conservation. Furthermore, recent research has revealed the existence of a secretory transmission mechanism in HAV, wherein the virus spreads through blood in a quasi-enveloped form, expanding traditional understanding of viral transmission. These unique molecular characteristics provide an important foundation for understanding HAV biological properties and developing antiviral strategies.

### 4.2 Epidemiology of HAV

The epidemiological characteristics of HAV demonstrate significant temporal-spatial variations and regional heterogeneity globally (Vaughan et al., 2014; Zufan et al., 2024). According to Global Burden of Disease Study (GBD) 2019 data, although global hepatitis A incidence and mortality demonstrated declining trends between 1990 and 2019, substantial inter-regional disparities persist (Levican et al., 2023). Low-income countries, due to poor sanitation conditions, remain hyperendemic regions, where most children acquire immunity through asymptomatic infection during early childhood; conversely, high-income countries, following improvements in sanitation, have transitioned from hyperendemic to hypoendemic status, yet with increased proportions of susceptible adult populations, resulting in greater numbers of clinical cases with more severe manifestations.

Regarding transmission routes, HAV spreads primarily through the fecal-oral route, including foodborne, waterborne, and close contact transmission. Recent studies indicate that important shifts in HAV transmission patterns have occurred in the vaccine era. Between 2016 and 2020, unprecedented person-to-person transmission outbreaks were reported across multiple U.S. states, predominantly affecting high-risk populations including homeless individuals, substance users, and men who have sex with men (MSM) (Bose et al., 2015). Population susceptibility distribution exhibits distinct age-related characteristics (Zufan et al., 2024). In hyperendemic regions, infection rates are highest among children under five years, though most infections are asymptomatic or mild; with increasing age, the proportion of naturally immune populations can exceed 90%. In hypoendemic regions, adolescents and adults remain susceptible due to lack of natural exposure opportunities, and once infected, typically present with classic clinical manifestations including jaundice and hepatic dysfunction (Narayanan et al., 2019; Poovorawan et al., 2025). Long-term surveillance data from Taiwan demonstrate that vaccination effectively alters population antibody seroprevalence distribution; populations born after 1995, having been incorporated into routine immunization programs, show significantly higher seropositivity rates compared to unvaccinated age-matched counterparts (Coudray-Meunier et al., 2014).

Implementation of vaccination strategies has profoundly transformed HAV epidemiological characteristics. Countries incorporating vaccine into routine childhood immunization have not only directly reduced infection rates in target populations but also protected unvaccinated elderly populations through herd immunity effects (Poovorawan et al., 2025). However, global vaccine coverage remains uneven, with accumulation of susceptible adult populations in some countries posing outbreak risks. Additionally, globalization and international travel accelerate transnational viral spread, placing hypoendemic regions under continuous threat of outbreaks triggered by imported cases. Future prevention and control strategies require expansion of vaccination coverage alongside strengthened surveillance and targeted interventions for high-risk populations.

### 4.3 The infection and pathogenic mechanisms of HAV

The infection process of HAV initiates with specific binding to host hepatocytes. The virus reaches the hepatic sinusoidal Disse space through the portal venous system and enters cells via receptor binding on the hepatocyte basal membrane (Ma et al., 2022). Hepatitis A virus cellular receptor 1 (HAVCR1), also known as T cell immunoglobulin and mucin domain-containing receptor 1 (TIM-1), represents the currently confirmed functional receptor for HAV. However, recent studies indicate that TIM-1 is not the sole cellular entry factor; HAV may enter cells through multiple receptor- or cofactor-mediated endocytic pathways, including integrins and asialoglycoprotein receptors (Zhang et al., 2018).

HAV exists in two infectious virion forms: naked virions detected in feces and quasi-enveloped virions detected in blood (Costafreda et al., 2020). Quasi-enveloped virions represent unique products of HAV hijacking host cell membrane trafficking systems. Following viral replication in hepatocytes, capsid proteins VP2 and VP1-pX interact with the endosomal sorting complex required for transport (ESCRT) and auxiliary proteins such as ALIX, loading capsids into intraluminal vesicles of multivesicular bodies (MVBs). Upon MVB fusion with the plasma membrane, virions are released into blood in the membrane-coated eHAV form. eHAV can evade immune recognition, enabling viral dissemination within the host (Li et al., 2004). When eHAV enters the biliary system with bile, bile acids remove its quasi-envelope, forming naked virions that are excreted with feces to complete the transmission cycle.

Following HAV infection of hepatocytes, viral RNA is released into the cytoplasm through endosomal acidification-triggered capsid conformational changes. The viral genome utilizes type III internal ribosome entry site (IRES) to initiate cap-independent translation, synthesizing a polyprotein precursor that is cleaved by viral proteases into structural and non-structural proteins (Deboosere et al., 2012). The pathogenic mechanism of liver injury primarily originates from host immune responses rather than direct viral cytotoxicity. HAV effectively suppresses type I interferon induction by encoding proteases 3ABC and 3CD to cleave mitochondrial antiviral signaling protein (MAVS) and TRIF signaling molecules, thereby evading innate immune surveillance (Yin et al., 2026). However, the virus cannot completely escape immune responses; plasmacytoid dendritic cells can recognize eHAV and produce type I interferons. Regarding adaptive immunity, virus-specific cytotoxic T lymphocytes (CTLs) have been traditionally considered the principal effector cells mediating hepatocellular injury, capable of lysing infected cells upon recognition of viral antigens. Recent research has revealed more complex mechanisms of HAV-associated liver injury: natural killer (NK) cells, natural killer T cells (NKT cells), and non-HAV-specific CD8+ T cells all participate in the liver injury process. Furthermore, MAVS signaling-mediated intrinsic apoptosis of infected hepatocytes has been observed in animal models as an additional cause of liver injury (Sharma et al., 2011).

Host genetic factors play important roles in disease severity. Studies have identified associations between TIM-1 gene polymorphisms and severe hepatitis risk; individuals carrying long-form TIM-1 alleles demonstrate enhanced cytotoxic activity of NKT cells against HAV-infected cells. Defects in the interleukin-18 binding protein (IL-18BP) gene can lead to unregulated IL-18, activating NK cell-mediated killing of both infected and uninfected hepatocytes and triggering fulminant hepatic failure (Li et al., 2004; Yin et al., 2026). These mechanisms are not mutually exclusive and may individually or synergistically participate in liver injury development under different clinical conditions.

### 4.4 The diagnosis, treatment, and prevention of HAV

Laboratory diagnosis of HAV relies on serological and molecular biological methods. Serological testing constitutes the cornerstone of clinical diagnosis, among which anti-HAV IgM antibody detection represents the gold standard for confirming acute HAV infection, detectable upon symptom onset and persisting for 4–6 months (Hu et al., 2020). Total anti-HAV antibody (HAVT) or IgG antibody testing is employed to assess immune status and history of past infection. In recent years, automated detection platforms such as chemiluminescent microparticle immunoassay (CMIA) have improved detection sensitivity and specificity. For molecular diagnosis, real-time quantitative RT-PCR technology has been widely applied for HAV RNA detection, enabling early and rapid diagnosis in blood and fecal samples. Application of next-generation sequencing technology has enabled whole-genome sequencing, providing precise tools for outbreak source tracing and viral evolution research (Vilà et al., 2004). Furthermore, development of dried blood spot (DBS) technology has facilitated sample preservation and transportation in remote areas, with serological and molecular detection sensitivity and specificity exceeding 90%.

Regarding treatment, no specific antiviral agents against HAV currently exist. Most acute self-limited HAV infections require only supportive care, including rest, adequate hydration, and nutritional support. For patients with severe cholestasis, cholestyramine, rifampicin, or ondansetron may be selected to alleviate pruritus; refractory pruritus rarely necessitates plasma exchange. For severe patients developing acute liver failure (ALF), transfer to intensive care units with close monitoring at liver transplantation centers is indicated. Although King’s College criteria may be utilized for assessing transplantation indications, their predictive value in HAV-associated ALF is limited, and dynamic prognostic models may be more instructive.

Prevention and control measures center on vaccination (Stuurman et al., 2017). Two vaccine types are currently available: inactivated and live-attenuated vaccines, both exhibiting favorable safety profiles and immunogenicity. WHO has formulated differentiated vaccination strategies based on national endemicity levels: hyperendemic regions rely primarily on natural immunity acquired through childhood exposure; mesoendemic regions recommend incorporating vaccine into routine childhood immunization programs; hypoendemic regions advise targeted vaccination of high-risk populations. The standard immunization schedule comprises 2–3 doses with intervals of at least 6 months, though single-dose vaccination has proven effective and been adopted in multiple countries (Fraisse et al., 2011). Vaccination not only directly protects recipients but also indirectly reduces infection risk in unvaccinated populations through herd immunity, significantly decreasing population-wide incidence and disease burden in high-risk groups.

Post-exposure prophylaxis represents an important means of controlling clustered outbreaks (Vilà et al., 2004). For unvaccinated exposed individuals (over 12 months of age), inactivated vaccine should be administered within 2 weeks of exposure. For immunocompromised individuals or those unable to receive vaccination within 14 days, immune globulin (IGIM) may be combined to provide passive immunoprotection. Vaccination coverage remains to be improved among high-risk populations including men who have sex with men, returning international travelers, homeless individuals, and intravenous drug users. Additionally, non-pharmaceutical interventions such as strengthened food safety supervision, improved sanitation facilities, and hand hygiene education remain fundamental control measures (Fraisse et al., 2011). Through multidisciplinary team collaboration, including infectious disease specialists, gastroenterologists, nurses, pharmacists, and public health professionals, patient prognosis can be significantly improved and epidemic transmission effectively controlled.

## 5 Other viruses

### 5.1 Hepatitis E virus

Hepatitis E virus (HEV) represents the sole member of the genus Orthohepevirus within the family Hepeviridae, a single-stranded positive-sense RNA virus (Ju and Ding, 2019). The genome spans approximately 7.2 kb and contains three open reading frames (ORFs): ORF1 encodes non-structural proteins comprising functional domains including methyltransferase, papain-like protease, helicase, and RNA-dependent RNA polymerase, responsible for viral replication; ORF2 encodes the capsid protein containing neutralizing epitopes that can self-assemble into virus-like particles (VLPs), inducing protective immunity; ORF3 encodes a small phosphoprotein involved in virion assembly, budding, and apical release in polarized hepatocytes. Recent studies have revealed that genotype 1 HEV uniquely expresses ORF4 protein, which can enhance viral replication capacity. HEV exists in two forms: naked virions (nHEV) and quasi-enveloped virions (eHEV), the latter hijacking host endomembrane transport systems for release from hepatocytes via multivesicular body (MVB) pathways, carrying host cell membrane components that enable immune evasion (Kenney and Meng, 2019; Gholizadeh et al., 2023).

Genotypes 3 and 4 predominate in European, North American, and East Asian regions, presenting as sporadic foodborne transmission; immunocompetent individuals typically manifest self-limited acute hepatitis, whereas solid organ transplant recipients, HIV-infected patients, and those with hematologic malignancies may progress to chronic infection, with approximately 60% of chronic infections developing cirrhosis (Lenggenhager and Weber, 2017). Recent research also demonstrates HEV hepatotropism extends beyond the liver, with invasion of the nervous system and kidneys causing Guillain-Barré syndrome and acute kidney injury (Wang et al., 2025).

Regarding infection and pathogenic mechanisms, following oral ingestion HEV traverses the intestinal barrier to enter the bloodstream, accessing hepatocytes through receptor-mediated endocytosis (Ju and Ding, 2019; Gholizadeh et al., 2023). Emerging research suggests epidermal growth factor receptor (EGFR) may serve as an accessory receptor for viral entry. Following replication, ORF2 promotes capsid assembly, while ORF3 interacts with ESCRT pathways to mediate viral budding release from MVBs. Hepatocellular injury mechanisms involve both direct viral cytotoxicity and host immune responses: ORF2 protein can directly impair mitochondrial function and induce endoplasmic reticulum stress; HEV-specific cytotoxic T lymphocytes, natural killer cells, and cytokine storms participate in hepatocyte injury; viral proteins additionally suppress interferon signaling pathways to evade innate immunity.

For treatment, immunocompetent acute infections receive primarily supportive care; chronic infections are treated with ribavirin monotherapy (600–1000 mg/day for 3–12 weeks), with approximately 80% of patients achieving sustained virologic response. For ribavirin-intolerant or non-responsive patients, pegylated interferon-α therapy may be considered (Treagus et al., 2021). Fulminant hepatitis or end-stage cirrhosis patients require liver transplantation evaluation.

Prevention strategies include improving drinking water sanitation and avoiding consumption of undercooked animal-derived foods. Hecolin vaccine represents the only currently licensed recombinant HEV vaccine, containing truncated p239 capsid protein, administered in three-dose (0, 1, 6 months) or two-dose (0, 1 month) regimens, demonstrating protective efficacy of 95.5%–100% in healthy populations and favorable efficacy in HBsAg-positive individuals (Wang et al., 2025). Vaccination during pregnancy shows good safety profiles and can protect neonates through transplacental antibodies. WHO recommends emergency vaccination in high-endemic regions, with two-dose rapid regimens applicable for outbreak control.

### 5.2 Hepatitis D virus

Hepatitis D virus (HDV) is a defective negative-strand RNA virus belonging to the genus Deltavirus, whose replication is strictly dependent on hepatitis B virus (HBV)-provided envelope proteins (HBsAg). Viral particles measure approximately 36–43 nm in diameter, with a genome consisting of a closed circular single-stranded RNA approximately 1.7 kb in length—the smallest known animal virus infecting humans (Stockdale et al., 2020). Eight genotypes have been confirmed to date: genotype 1 demonstrates global distribution, genotype 2, 4 is predominantly found in Asia, genotype 3 in South America, and genotypes 5–8 originate from Africa. Approximately 4.5% of HBsAg-positive individuals globally (12–20 million people) are coinfected with HDV, primarily distributed in hyperendemic regions including the Middle East, Central Asia, North Africa, and Pacific islands; HBV-positive individuals in Europe and North America show infection rates of approximately 3%–6%. Transmission routes are identical to HBV, including blood, bodily fluids, and vertical mother-to-child transmission. Intravenous drug users, hemodialysis patients, men who have sex with men, and sexual partners of HBV carriers constitute high-risk populations. Two infection patterns exist: coinfection with HBV (simultaneous acquisition) or superinfection (HDV infection of established HBV carriers). Superinfection more readily leads to chronicity, with approximately 90% progressing to chronic hepatitis D (CHD) (Urban et al., 2021).

Regarding infection and pathogenic mechanisms, HDV enters hepatocytes through binding of HBV envelope protein preS1 domain to the sodium-taurocholate cotransporting polypeptide (NTCP) receptor, a process that can be blocked by novel therapeutic agents (Asif and Koh, 2022; Khalfi et al., 2023). Diagnosis relies on serological and molecular biological methods. All HBsAg-positive individuals should be screened for anti-HDV (total antibodies or IgG), with positive cases requiring real-time quantitative PCR detection of HDV RNA to confirm active infection. In recent years, automated detection platforms (such as Cobas systems) have improved standardization, though detection performance for African genotypes 5–8 requires optimization (Asif and Koh, 2022).

For treatment, pegylated interferon-α PEG−IFNα was previously the sole option, but sustained virologic response rates reached only 20%–30% with significant side effects. In 2023, the European Union approved Bulevirtide (2 mg/day subcutaneous injection), an NTCP receptor inhibitor that blocks viral entry; monotherapy for 48 weeks enables 45%–78% of patients to achieve HDV RNA decline ≥2 log or ALT normalization, with combination PEG-IFNα improving response rates (Stockdale et al., 2020; Asif and Koh, 2022). Other investigational agents include the prenylation inhibitor Lonafarnib (inhibiting L-HDAg prenylation to block viral assembly), RNA interference molecule Elebsiran (silencing HBV RNA), and monoclonal antibodies (such as Tobevibart). Prevention centers on HBV vaccination; all HBV-uninfected individuals should be vaccinated, while infected individuals should undergo regular HDV screening with strengthened blood safety management and needlestick injury protection (Hoblos and Kefalakes, 2023).

### 5.3 Human cytomegalovirus

The clinical association between human cytomegalovirus (HCMV) and viral hepatitis is manifested primarily in differential diagnosis and hepatic injury. Clinically, HCMV is diagnosed almost exclusively through laboratory testing, as its clinical manifestations often resemble viral hepatitis, necessitating differentiation through detection of anti-HCV, anti-HIV, hepatitis B antigens, and hepatitis A and E markers (Shang and Li, 2024). HCMV infection can cause elevated transaminases, cholestasis, and hepatocellular necrosis, presenting as atypical hepatitis syndrome. Studies indicate that both congenital and acquired HCMV infections can induce hepatic injury, including hepatomegaly, hepatitis, cholestatic liver disease, and even cirrhosis, with mechanisms potentially related to HCMV-IE2 protein inhibition of hepatocyte proliferation, promotion of apoptosis, and interference with hepatocyte maturation.

Regarding molecular characteristics, HCMV belongs to the Betaherpesvirinae subfamily and represents one of the largest known human viruses (Griffiths et al., 2015; Lee et al., 2023). Its double-stranded DNA genome spans approximately 230 kb, comprising unique long (UL) and unique short (US) segments, encoding approximately 200 proteins. The viral structure consists, from exterior to interior, of lipid envelope, tegument, and icosahedral nucleocapsid. Envelope glycoproteins gB (UL55) and gH (UL75) mediate viral binding and fusion with host cells; tegument protein pp65 (UL83) possesses significant antigenicity and inhibits IRF-3-mediated type I interferon responses, serving as a critical factor for immune evasion. Non-structural proteins such as IE1 and IE2 function as immediate-early proteins regulating viral gene expression, with IE2 acting as the master regulator of viral infection progression (Fernández-Moreno et al., 2021).

Epidemiologically, HCMV demonstrates global distribution. Seropositivity rates among women of childbearing age range from 40%–83% in developed countries and approach 100% in developing countries. Congenital infection incidence reaches approximately 0.6%–0.7% in developed countries (approximately 40,000 cases annually in the United States) and 1%–5% in developing countries. Previous research screening approximately 515,646 infants globally revealed an overall congenital HCMV prevalence of 0.67%, with low-income countries demonstrating three-fold higher prevalence than high-income countries. High-risk populations include organ transplant recipients, HIV-infected individuals, and children with congenital immunodeficiencies (Fernández-Moreno et al., 2021). Transmission routes encompass vertical mother-to-child transmission, blood products, and close bodily fluid contact.

Infection and pathogenic mechanisms are complex. HCMV enters cells through gB/gH glycoprotein binding to multiple cell surface receptors, followed by endocytosis and membrane fusion, with nucleocapsids entering the nucleus via microtubules (Fernández-Moreno et al., 2021). Hepatocellular injury mechanisms involve both direct cytotoxic effects and indirect immune-mediated damage: HCMV can directly lyse hepatocytes; CD8+ cytotoxic T lymphocytes and proinflammatory cytokines serve as primary mediators of immunopathology. Prospective studies by Yokota suggest that HBV and HCV infections may indirectly influence HCMV infection susceptibility through effects on host immune status or hepatocellular environment (Abdalla et al., 2020). Although direct virus-virus interaction mechanisms remain incompletely elucidated, HCMV coinfection with hepatitis viruses in immunocompromised patients can significantly exacerbate hepatic pathological injury.

Diagnosis relies on serology (IgM, IgG, IgG avidity) and nucleic acid testing (HCMV DNA quantitative PCR). In 2017, the novel terminase inhibitor letermovir was approved for post-hematopoietic stem cell transplantation prophylaxis, inhibiting pUL56-mediated viral DNA cleavage and packaging without myelosuppression; in 2021, the UL97 protein kinase inhibitor maribavir was approved for resistant/refractory HCMV disease. Immunoglobulin combined with antiviral agents is used for severe cases (Abdalla et al., 2020; Lee et al., 2023). No approved vaccines currently exist; multiple candidate vaccines including glycoprotein B subunit vaccines, pp65 nanoparticle vaccines, replication-deficient Towne strain vaccines, and mRNA vaccines are undergoing clinical trials.

### 5.4 Epstein-Barr virus

The clinical association between Epstein-Barr virus (EBV) and viral hepatitis is manifested primarily in differential diagnosis and hepatic injury. Clinically, EBV infection commonly presents with non-hepatic-specific systemic symptoms; approximately 90% of infectious mononucleosis (IM) patients develop mild subclinical hepatitis, only 10%–15% demonstrate significant hepatic dysfunction, and few progress to fulminant hepatic failure. Due to overlapping clinical manifestations and laboratory findings between EBV hepatitis and viral hepatitis (such as hepatitis A, B, and E), differentiation requires serological testing (anti-HAV, HBsAg, anti-HCV, HEV RNA/antibody) (Roca-Millan et al., 2021). Liver biopsy reveals characteristic sinusoidal lymphocytic infiltration and EBER-ISH positive cells, with CD8+ T cytotoxic responses representing the principal mechanism of hepatic injury.

Regarding molecular characteristics, EBV belongs to the Gammaherpesvirinae subfamily and is one of the known human oncogenic viruses. Its double-stranded DNA genome spans approximately 172 kb, encoding over 80 open reading frames (Iizasa et al., 2022). Viral particles comprise a pleomorphic nucleocapsid, tegument, and envelope, with surface glycoproteins gp350/220 mediating binding to CD21 (CR2) receptors, and gp42 engaging HLA class II molecules and CD94-NKG2A to promote membrane fusion and cellular entry.

Epidemiologically, EBV demonstrates global distribution, with approximately 95% of the world’s population having been infected. Infection rates increase with age; developed countries exhibit delayed primary infection age (approximately 50%–54% at 6–8 years, reaching 90% by 18–19 years), predisposing to symptomatic infectious mononucleosis, whereas developing countries experience earlier infection, often asymptomatic (Iizasa et al., 2022). Transmission occurs primarily through saliva contact (such as kissing, shared utensils), with additional routes including blood and organ transplantation. High-risk populations include adolescents, university students, organ transplant recipients, and HIV-infected individuals. In recent years, EBV seropositivity rates in developed countries have declined (from 64% in 2010 to 52% in 2025 among U.S. university students), increasing the risk of IM development in uninfected adolescents (Mark et al., 2025).

Infection and pathogenic mechanisms are complex. EBV enters cells through gp350 binding to B cell surface CD21, followed by endocytosis, forming circular episomes and establishing lifelong latency. Hepatocellular injury mechanisms involve both direct cytotoxic effects and immune-mediated damage. Virus-infected B cells and T cells infiltrate hepatic tissue, releasing cytokines IFN−γ,TNF−α and perforin/granzyme; EBV-specific cytotoxic T lymphocytes recognize and kill infected cells; RFA-A gene activation induces apoptosis and necrosis (Roca-Millan et al., 2021; Yang et al., 2023). EBV itself does not directly damage hepatocytes but indirectly causes hepatocellular injury through lymphocytic infiltration and inflammatory responses, clinically manifesting as markedly elevated hepatic enzymes, elevated conjugated bilirubin, and in severe cases progression to cholestatic hepatitis or even hepatic failure.

Diagnosis relies on serology and molecular testing. Serological testing includes anti-VCA IgM (acute infection marker), anti-VCA IgG (past infection marker), anti-EBNA IgG (post-infectious mononucleosis marker, appearing months after acute infection), and heterophile antibodies (Monospot test). Real-time quantitative PCR for EBV DNA viral load is used for diagnosing acute infection, monitoring immunosuppressed patients (such as post-transplant), and confirming EBV-associated lymphoproliferative disorders (Yang et al., 2023). Liver biopsy EBER *in situ* hybridization detection represents the diagnostic gold standard.

For treatment, most EBV-associated hepatic injury is self-limited, requiring only supportive care and symptomatic management (hydration, nutritional support, hepatoprotective agents) (Roca-Millan et al., 2021; Mark et al., 2025). Corticosteroids are indicated for severe airway obstruction or thrombocytopenia, though evidence remains limited. Antiviral agents are not employed, as they are ineffective against latent infection. Moderna mRNA-1189 and Moderna mRNA-1195 preventive vaccines currently in phase III clinical trials aim to block primary infection or attenuate IM symptoms. EBV glycoprotein gp350 vaccines can induce neutralizing antibodies, preventing asymptomatic infection and infectious mononucleosis. Additional strategies include therapeutic vaccines targeting LMP1/LMP2 to induce specific T cell responses.

### 5.5 Herpes simplex virus

The clinical association between herpes simplex virus (HSV) and viral hepatitis is manifested primarily in acute hepatic injury and differential diagnosis. Clinically, HSV infection is diagnosed almost exclusively through laboratory testing, as its clinical manifestations often overlap with viral hepatitis, necessitating differentiation through detection of hepatitis A, B, C, and E markers. HSV-1/2 infection can cause acute necrotizing hepatitis, with severe cases progressing to acute liver failure (ALF), particularly in immunosuppressed individuals, especially following hematopoietic stem cell or solid organ transplantation (Cole, 2020). Typical cases demonstrate markedly elevated transaminases, cholestasis, and coagulopathy; liver biopsy reveals extensive coagulative necrosis with positive HSV immunostaining. Early diagnosis is challenging because HSV hepatitis is rare (accounting for less than 1% of ALF etiologies) and characteristic mucocutaneous lesions are absent in early stages (Chang et al., 2024; Dutton et al., 2025).

Epidemiologically, HSV demonstrates global distribution with extremely high human infection rates. Global estimates in 2020 indicated approximately 3.8 billion individuals aged 0–49 years were infected with HSV-1 (prevalence 64.2%), predominantly oral infections; 520 million individuals aged 15–49 years were infected with HSV-2 (prevalence 13.3%), primarily genital infections. HSV-2 infection exhibits marked gender and regional disparities, with higher infection rates in females (17.0%) than males (9.7%), and the heaviest burden in African regions. Transmission routes include close contact (oral-oral, genital-genital), vertical mother-to-child transmission, and organ transplantation (Chang et al., 2024).

Infection and pathogenic mechanisms are complex. HSV enters cells through gB/gD binding to cell surface receptors (HVEM, nectin-1, etc.), followed by endocytosis; after tegument and envelope removal, the nucleocapsid enters the nucleus (Cole, 2020). Hepatocellular injury mechanisms involve both direct viral cytotoxicity and immune-mediated damage: viral lysis of hepatocytes, complement system activation, and apoptosis induction; concurrently, CD8+ T cells, NK cells, and proinflammatory cytokines TNF−α,IL−6 participate in immunopathological injury.

Diagnosis relies on serology (IgM, IgG) and nucleic acid testing (HSV DNA real-time PCR). PCR detection of viral DNA in blood or tissue represents the diagnostic gold standard; positive HSV immunohistochemical staining in liver biopsy confirms diagnosis. For treatment, acyclovir/valacyclovir constitute first-line antiviral agents, requiring initiation within 4 days of ALF symptom onset to reduce mortality (from 80%–90% to 50%–55%). Foscarnet is reserved for acyclovir-resistant cases (resistance rates reaching 5% in immunosuppressed patients). Therapeutic plasma exchange (TPE) can clear inflammatory factors and viral load as a supportive therapeutic modality (Hua and Wakimoto, 2019). Liver transplantation represents the definitive treatment for end-stage hepatic failure but requires timely evaluation before severe complications develop. No licensed vaccines currently exist; gD protein subunit vaccines and mRNA vaccines are under development.

### 5.6 Yellow fever virus

The clinical association between yellow fever virus (YFV) infection and viral hepatitis is manifested primarily in differential diagnosis and acute hepatic injury. Clinically, yellow fever is diagnosed almost exclusively through laboratory testing, as its clinical manifestations (fever, jaundice, hemorrhage) often overlap with hepatitis A, B, C, and E, necessitating differentiation through serological detection (Waggoner et al., 2018). YFV infection causes characteristic acute hepatitis, presenting with markedly elevated transaminases, high direct bilirubinemia, and coagulopathy. Severe patients progress to acute liver failure (ALF) with mortality rates reaching 40%–50%. Liver biopsy reveals extensive coagulative necrosis, microvesicular steatosis, mitochondrial structural disruption, and E protein deposition. Recent studies have identified hepatic enzyme rebound phenomena occurring 2–6 months post-recovery in some patients, suggesting immune-mediated persistent hepatic injury may participate in disease progression (Beasley et al., 2015).

Regarding molecular characteristics, yellow fever virus belongs to the genus Flavivirus within the family Flaviviridae, a single-stranded positive-sense RNA virus (Beasley et al., 2015). The genome spans approximately 11 kb, with a type I cap structure at the 5’ end and no polyadenylate tail at the 3’ end. The virus encodes three structural proteins: capsid protein (C), membrane protein precursor (prM), and envelope glycoprotein (E); and seven non-structural proteins (NS1, NS2A, NS2B, NS3, NS4A, NS4B, NS5).

Epidemiologically, yellow fever is endemic to 44 countries in tropical Africa and Central and South America. WHO estimates 63,000–158,000 severe infections annually, causing 29,000–75,000 deaths, with over 90% occurring in Africa, where the Democratic Republic of Congo and Nigeria bear the heaviest burden. In 2018, approximately 109,000 severe infections and 51,000 deaths occurred globally. Three transmission cycles exist: jungle cycle (mosquito-non-human primate-human, predominant South American pattern), urban cycle (mosquito-human-mosquito, predominant African epidemic pattern), and intermediate cycle (African savanna). Aedes aegypti and Haemagogus species serve as principal vectors (Kleinert et al., 2019). High-risk populations include unvaccinated travelers, forest workers, infants and young children, and non-immune urban residents.

Infection and pathogenic mechanisms are complex. The virus enters cells through E protein binding to cell surface receptors, followed by clathrin-mediated endocytosis; low pH environments trigger E protein conformational changes inducing membrane fusion and nucleocapsid release into the cytoplasm (Waggoner et al., 2018; McArthur et al., 2020). Extensive endothelial cell damage leads to increased vascular permeability, disseminated intravascular coagulation, and multi-organ dysfunction, constituting the principal pathological basis for hemorrhagic tendency and shock.

Diagnosis relies on serological and molecular biological methods. Serum IgM antibody detection represents the principal means of confirming acute infection, detectable 5 days after disease onset and persisting for months; real-time quantitative RT-PCR for viral RNA in blood, urine, or tissue demonstrates high sensitivity and specificity, applicable for early diagnosis (McArthur et al., 2020). For treatment, no specific antiviral agents currently exist; clinical management is primarily supportive, including fluid replacement, nutritional support, hepatoprotective agents, and coagulation function correction (Barrett and Monath, 2003). The World Health Organization Eliminate Yellow Fever Epidemics (EYE) strategy aims to vaccinate 1.3 billion high-risk populations by 2026, combined with vector control and cross-border surveillance, striving to interrupt yellow fever transmission chains.

## 6 Conclusions and perspectives

Viral hepatitis represents a heterogeneous disease spectrum caused by a diverse array of DNA and RNA viruses, with genome sizes spanning nearly two orders of magnitude from 1.7 kb to 235 kb. Despite marked differences in viral architecture, replication strategies, and host tropism, these pathogens converge on common pathogenic pathways—direct cytotoxicity and/or immune-mediated injury—to produce a remarkably broad clinical spectrum ranging from subclinical infection to fulminant hepatic failure, and from acute self-limited hepatitis to chronic progressive disease. This biological and clinical diversity underscores the substantial global burden of viral hepatitis and the complexity of its clinical management. Beyond the classical hepatotropic viruses (HBV, HCV, HAV, HEV, and HDV), emerging and opportunistic pathogens including CMV, EBV, HSV, and YFV increasingly contribute to severe hepatic injury, particularly in immunocompromised hosts and endemic settings. The overlapping clinical phenotypes among these agents necessitate a paradigm of precise differential diagnosis anchored in multiplex serological profiling and nucleic acid amplification technologies (Zhao et al., 2016; Seto et al., 2020). Looking forward, several priorities warrant focused attention. First, the integration of next-generation sequencing, artificial intelligence-assisted imaging, and multiplex molecular diagnostics promises to revolutionize early detection and real-time surveillance of both established and emerging hepatotropic viruses. Second, therapeutic innovation must extend beyond the current direct-acting antiviral and nucleos(t)ide analogue regimens to address persistent virological reservoirs—most notably HBV cccDNA and HCV reinfection cascades—through combinatorial strategies involving gene editing, therapeutic vaccines, and host-targeting agents. Third, universal vaccination coverage, harm-reduction programs, and strengthened blood safety remain indispensable pillars for primary prevention, particularly in resource-limited regions where the disease burden remains highest. Finally, achieving the WHO 2030 elimination targets for viral hepatitis will require coordinated global efforts to bridge diagnostic and treatment gaps, eliminate stigma, and ensure equitable access to care across diverse healthcare systems (Koff, 1974). Through sustained multidisciplinary collaboration spanning virology, immunology, hepatology, and public health, the transition from disease control to elimination of viral hepatitis as a public health threat represents an attainable, albeit demanding, objective for the coming decade.

Epidemiological characteristics demonstrate marked regional distribution and population aggregation tendencies (Burns and Thompson, 2014). HBV and HDV exhibit specific endemic patterns in low-intermediate and high-endemic regions; HAV and HEV manifest waterborne and foodborne transmission; YFV relies upon mosquito vectors for jungle and urban cycles (Stasi et al., 2019; Sitnik et al., 2021). Global burden of disease studies demonstrate that differentiated implementation of vaccination strategies has significantly transformed the epidemiological characteristics of HAV and HBV, whereas foodborne HEV outbreaks, high HDV incidence among HBV carriers, and YFV diffusion during urbanization processes indicate that prevention and control strategies require region-specific adaptation (Hussain et al., 2011).

Research on infection and pathogenic mechanisms has revealed the high complexity of host-virus interactions (Zhang et al., 2024). All viruses have evolved sophisticated immune evasion strategies: HAV and HEV escape neutralizing antibodies through quasi-enveloped forms; HBV maintains persistent infection through cccDNA; CMV and EBV suppress interferon signaling pathways and antigen presentation through specific proteins; while HCV and HDV directly disrupt apoptotic pathways through viral polymerases and accessory proteins (Piconese et al., 2018; Quirino et al., 2024). These mechanisms not only determine clinical outcomes but also provide molecular targets for developing targeted therapeutics (Larrubia et al., 2008).

At the diagnostic, therapeutic, and preventive levels, advances in serological and molecular biological technologies have rendered precise diagnosis achievable, while antiviral therapy has evolved from non-specific immune modulation to precision interventions targeting viral replication (Nguyen and Keeffe, 2009; Dunkelberg et al., 2014). Targeted agents including pegylated interferon, nucleos(t)ide analogues, NS3/4A protease inhibitors, NS5A inhibitors, and polymerase inhibitors have significantly improved prognoses for chronic viral hepatitis, while novel molecularly targeted drugs such as bulevirtide and letermovir have brought breakthroughs for HDV and CMV infections (Chan et al., 2022). Vaccines, as the most successful public health intervention, have effectively controlled transmission of HAV, HBV, and YFV, whereas development of prophylactic vaccines for HCV, HDV, and CMV remains unfinished business (Chen et al., 2017; Cooke et al., 2019). Future research should focus on deep mechanisms of virus-host interactions, development of novel antiviral targets, and design of broad-spectrum antiviral vaccines, ultimately achieving the ambitious goal of eliminating viral hepatitis.
